# The Interstitial Collagenases MMP13 and MMP14 Are Dispensable for the Early Onset of Skin Morphogenesis but Modulate Endochondral Ossification

**DOI:** 10.1016/j.ajpath.2026.04.018

**Published:** 2026-05-15

**Authors:** Birgit Bouriette, Luca Gaessler, Jan Zamek, Joy Steinkamp, Pitter F. Huesgen, Bent Brachvogel, Cornelia Mauch, Paola Zigrino

**Affiliations:** ∗ Department of Dermatology and Venereology, University of Cologne, Faculty of Medicine and University Hospital Cologne, Cologne, Germany; † Faculty of Biology and CIBSS–Centre for Integrative Biological Signaling Studies, University of Freiburg, Freiburg, Germany; ‡ Department of Pediatrics and Adolescent Medicine, Experimental Neonatology, University of Cologne, Faculty of Medicine and University Hospital Cologne, Cologne, Germany; § Cologne Excellence Cluster on Cellular Stress Responses in Aging-Associated Diseases (CECAD), University of Cologne, Cologne, Germany; ¶ Center for Molecular Medicine Cologne (CMMC), University of Cologne, Cologne, Germany; || Department of Dermatology, Ruhr University Bochum, Bochum, Germany

## Abstract

Connective tissue remodeling is vital for organ development and tissue stability. Matrix metalloproteinases (MMPs), especially MMP14 and MMP13, are important collagenases that break down interstitial collagen in bone and skin. In mice, the loss of MMP14 results in perinatal death and severe skeletal defects, whereas *Mmp13* deficiency causes transient bone defects that later resolve. Despite abundant dermal collagen, single knockouts usually do not display defects in skin formation, likely due to compensatory mechanisms. To examine their combined roles, mice lacking both enzymes were generated. These double knockout mice were viable at birth but rapidly developed severe symptoms resembling *Mmp14* deficiency, including growth retardation, wasting, and death within 3 weeks. Surprisingly, skin structure remained largely normal, aside from early subcutaneous fat loss also observed in the *Mmp14*-deficient mice, indicating that neither enzyme is essential for early dermal remodeling. In contrast, skeletal defects worsened, showing shortened long bones, delayed primary ossification, impaired collagen type I breakdown, and reduced vascular invasion. These findings indicate that MMP13 partly compensates for MMP14 during bone development, while both enzymes are unnecessary for postnatal dermal collagen remodeling. Because MMP mutations are linked to human skeletal dysplasia, fibrosis, and wound-healing issues, these results underscore the distinct roles of MMP14 and MMP13 in collagen turnover and highlight their potential importance in human bone and connective tissue disorders.

Remodeling of the extracellular matrix (ECM) is essential for tissue development, growth, and homeostasis. The dynamic balance between ECM synthesis and degradation maintains tissue integrity, while disruptions in this balance can lead to connective tissue disorders such as fibrosis, characterized by excessive collagen accumulation. Higher fibrillar collagen content is observed in organs that require mechanical strength, structural integrity, and resistance to stretching or deformation, such as skin and bone. In these organs, fibrillar collagens (types I, II, III, and V) are the most abundant, undergoing continuous remodeling to maintain their function in tissues over time.^1^

During this remodeling process, collagen catabolism is mediated by collagenases, including cysteine proteases and matrix metalloproteinases (MMPs). Among the MMPs, MMP8, MMP13, MMP14, and MMP16 have exhibited collagenolytic activity; MMP13 and MMP14 have been particularly well studied.^2^ The expression and function of MMP13 and MMP14 may be distinct during tissue morphogenesis, but there are also points of overlap, particularly in ECM remodeling and activation of other MMPs. Both MMP13 and MMP14 are collagenases that exhibit some specificity for the different fibrillar collagens. MMP13 cleaves fibrillar collagen types I, II, and III, with a preference for collagen type II. Also, MMP14 cleaves collagen types I and II, with type I as its primary substrate, and it acts on collagen type II and possibly collagen type III.^3^

*In vivo* genetic deletion of these proteases has shown that MMP13 is not crucial for skin development or homeostasis, as *Mmp13*-deficient mice exhibit normal epidermal structure and dermal composition.^4^ However, although skin repair generally occurs in these mice, an initial delay in re-epithelialization and wound contraction was observed in another model of *Mmp13* deficiency.^5^ In contrast, although *Mmp13* knockout mice are viable and fertile, the complete removal of *Mmp14* results in early death at around 3 weeks of age, yet these mice surprisingly exhibit no skin phenotype. They do not show defects in skin collagen remodeling at this early stage.^6^ A previous study reported that a few mice, which, for unknown reasons, survive past early death and reach approximately 100 days of age, develop progressive soft tissue fibrosis,^7^ consistent with impaired collagen-degrading activity. In support of the later role of MMP14 in skin remodeling during aging, induced deletion of *Mmp14* specifically in fibroblasts at postnatal ages led to collagen accumulation and a fibrosis-like skin disease due to impaired fibrillar collagen type I.^8^ The collagen remodeling activity mediated by MMP14 was required only for homeostatic turnover under steady-state conditions but not during tissue repair.^8^^,^^9^ Thus, MMP14 appears to be more critical for homeostasis in adult skin than for skin morphogenesis, in which several other enzymes, or the same MMPs, can likely compensate for its activity.

Both proteases are detected in an overlapping pattern during skeletal morphogenesis, and *in vivo* deletion studies of MMP13 and MMP14 in mice have revealed their critical, and in some cases complementary, roles during bone development. In bone tissue, MMP14 is detected in many cell types, including osteoclasts.^10^ However, it is also found in the perichondrium and periosteum during bone development.^11^ Further studies in reporter mice show that MMP14 expression in embryos is restricted to blood vessels, the cartilage primordium, and muscle tissues, suggesting a role in embryonic cartilage development.^12^ Indeed, it was shown that in osteoblasts, it modulates the PTH/PTH1R pathway in transition into subchondral bone.^13^ MMP13 is expressed in the skeleton by osteoblasts and hypertrophic chondrocytes^10^^,^^11^ and is notably found in primary ossification centers (POCs) and sites of cartilage-to-bone transition.^14^^,^^15^ In line with these expression patterns, mice deficient in *Mmp14* display pronounced skeletal abnormalities, including dwarfism, severe osteopenia, generalized arthritis, and shortened lifespan.^6^^,^^7^ Interestingly, despite severe skeletal phenotypes, *Mmp14*-null mice survive for a few postnatal weeks, suggesting compensatory mechanisms involving another protease.

In line with findings in mouse models, a human MMP14 mutation causes Winchester syndrome. This rare skeletal disorder shares many phenotypic traits with *Mmp14* knockout mice, including short stature, low bone density, arthritis, reduced adipose tissue, and craniofacial abnormalities.^16^^,^^17^ In addition, these patients display cutaneous features that are heterogeneous, including diffusely thickened, leathery skin and subcutaneous nodules.^18^

*Mmp13* deletion in mice results in delayed remodeling of hypertrophic cartilage at the growth plate, leading to skeletal development abnormalities that resolve over time; these mice remain fertile, however, and have a normal lifespan.^19^ Likewise, a point mutation in human MMP13 causes the Missouri variant of spondyloepimetaphyseal dysplasia, a developmental disorder marked by defective growth and modeling of the long bones. Notably, this condition tends to improve with age.^20^ Thus, although MMP13 and MMP14 were co-identified in key tissues such as bone and skin, deletion of either gene alone does not fully recapitulate the phenotypes expected from their expression profiles. Also, although the bone phenotypes are those observed in human diseases with mutations in these genes, in the skin, there may be functional redundancy.

Inactivation of both collagenases was used in the current study to identify whether MMP13 and MMP14 can compensate for each other’s role in collagen turnover during development. The findings reveal that, although these mice largely recapitulate the severe phenotype of *Mmp14* deficiency, they also exhibit additional defects not observed in the complete *Mmp14* knockout, including a lack of secondary ossification center formation and a substantial delay in POC development due to impaired collagenolysis.

## Materials and Methods

### Generation of the *Mmp13*/*Mmp14* Double Knockout Strain

Double-deficient *Mmp14/Mmp13* [double knockout (DKO)] mice were generated by crossing *Mmp13*-deficient mice^19^ with *Mmp14*-deficient mice,^6^ both on a C57BL/6 background. In detail, DKO mice were generated by crossing *Mmp13*^*+/–*^*/Mmp14*^*+/–*^ double heterozygotes, derived from *Mmp13*^*–/–*^ × *Mmp14*^*–/–*^ single-KO intercrosses. Hygiene monitoring of the facility’s mice was conducted quarterly in accordance with Federation of European Laboratory Animal Science Associations guidelines. Genotyping was performed by PCR amplification of the targeted allele using genomic DNA extracted from tail biopsy specimens.

For genotyping, the following primers were used: *Mmp14* knockout allele, 5′-GCTATGACTGGGCACAACAGA-3′ (forward) and 5′-CAAGGTGAGATGACAGGAGAT-3′ (reverse); *Mmp14* wild type (WT), 5′-ATAGCTCTAATCTTGCAGAG-3′ (forward) and 5′-GGCTCGGCAGAATCAAAGTG-3′ (reverse); *Mmp13* knockout, 5′-CCCAAACGAACTTAACTTACAG-3′ (forward) and 5′-GGTCTTCATCGCCTGGAC-3′ (reverse); and *Mmp13* WT, 5′-TGATGATGACGTTCAAGGAATTC-3′ (forward) and 5′-CTGGCAGCAAACTGCAATGT-3′ (reverse). Tails were incubated in lysis buffer (100 mmol/L Tris-HCl, 5 mmol/L EDTA, 0.2% SDS, 200 mmol/L NaCl, 200 μg/mL Proteinase K, pH 8) overnight at 55°C. DNA was precipitated with 10 μL of 3 mol/L sodium acetate and 1 volume of ethanol. After washing, the DNA was resolved in 100 μL of water.

The local veterinary authority approved the animal experiments (NRW authorization 50.203.2-K 37a, 20/05, 87-51.04.2010.A34, 84-02.04.2015.A516). WT embryo specimens from C57BL/6N mice (with the same genetic background as the *Mmps*-deficient mice) were kindly provided by Dr. Hisham Bazzi (CECAD, Medical Faculty, University of Cologne, Cologne, Germany; NRW authorization 81-02.04.2021.A130).

### Analysis of Blood Adiponectin, Leptin, and Glucose Levels in Mice

A blood drop from tails was used for glucose, leptin, and adiponectin quantification. Blood was analyzed by applying it to a GlucoMen sensor control strip (Code F1, Reference 24654, Lot 1130210187; A. Menarini Diagnostics, Division Berlin-Chemie AG, Berlin, Germany) and measured by using a GlucoMen PC (SN00251258; A. Menarini Diagnostics). Data are expressed in milligrams per deciliter. Calibration and handling were performed according to the manufacturer’s instructions.

For the quantification of adiponectin and leptin, whole blood was collected directly from the heart after death and centrifuged at 2000 × *g* for 30 minutes at 4°C. Separated serum was collected, frozen at –20°C, and analyzed by Mediagnost (Reutlingen, Germany).

### Cell Collagen Processing Assay

Type I fibrillar collagen degradation was assessed in a cell-based assay as described previously.^7^^,^^8^ Briefly, 24 multiwell plates were coated with a film of reconstituted rat tail tendon type I collagen fibrils. Isolated primary dermal fibroblasts from all mouse genotypes were seeded on top for 5 hours, washed in phosphate-buffered saline (PBS), and incubated for 5 days in serum-free Dulbecco’s modified Eagle’s medium [Life Technologies (Thermo Fisher Scientific), Darmstadt, Germany] with or without the addition of 10^−8^ mol/L tumor necrosis factor-α and GM6001 (5 μmol/L; Bio-Techne GmbH, Wiesbaden-Nordenstadt, Germany). After incubation, cells were removed with trypsin/EDTA (Life Technologies), and the collagen fibril film was stained with Coomassie Blue (Sigma-Aldrich, Taufkirchen, Germany). RNA extraction from cells [after 24 hours without or with phorbol 12-myristate 13-acetate (PMA), 100 nmol/L] or back skin, and RT-PCR or real-time quantitative PCR were performed as previously described.^8^

### Fixation and Decalcification of Long Bones

The skin of both hind legs of mice at postnatal day 14 (P14) was completely removed, and the legs were fixed in 4% paraformaldehyde for 2 days at 4°C. Legs were then transferred to a 0.5 mol/L EDTA buffer (pH 8) and incubated at 4°C under shaking conditions for at least 2 weeks. The EDTA solution was changed three to four times. After decalcification, the legs were briefly washed in PBS and then incubated in 40% ethanol for 1 hour and in 70% ethanol for 3 more hours at 4°C. Legs were further dehydrated in an ascending alcohol series using an automated vacuum tissue processor (ASP 300S; Leica Biosystems, Wetzlar, Germany) and finally embedded in paraffin. Tissue was sectioned at 7 μm using a Shandon Finesse Microtome (Thermo Fisher Scientific, Waltham, MA), collected on microscope slides (Gerhard Menzel GmbH, Braunschweig, Germany), and dried overnight at 37°C. Deparaffinization was performed by incubating the slides in xylene (20 minutes), followed by a graded alcohol series (1 minute each): isopropyl alcohol, 96% ethanol, 75% ethanol, and double-distilled water.

### Immunofluorescence and Histochemistry

Decalcified tissue of the murine femur was fixed in 4% neutral buffered formalin, dehydrated in an ascending alcohol series, and embedded in paraffin. Tissue sections (7 μm) were deparaffinized and rehydrated in a descending alcohol series before being stained with hematoxylin and eosin or analyzed by immunohistochemical methods after antigen retrieval with citrate buffer (pH 6; Dako Cytomation, Glostrup, Denmark). For Alcian Blue staining, deparaffinized sections were stained with Dako REAL hematoxylin (Dako Cytomation) for 5 minutes, washed with water, and stained in a 0.03% Alcian Blue solution for 20 minutes. After washing, counterstaining was performed by using 1% Eosin Y (Thermo Scientific, Runcorn, United Kingdom) for 4 minutes, followed by an ascending alcohol series (70% ethanol for 2 minutes, 90% ethanol for 2 minutes, 96% ethanol for 2 minutes, isopropyl alcohol twice for 2 minutes each, and xylene twice for 2 minutes each).

Alternatively, after deparaffinization, sections were stained with 0.001% Fast Green for 5 minutes. After being briefly rinsed in 1% acetic acid, sections were stained in 0.1% Safranin O for 5 minutes and then dehydrated in 96% ethanol, isopropyl alcohol, and xylene, each for 2 minutes. For tartrate-resistant acid phosphatase staining on sections, a kit was used and the manufacturer’s instructions were followed (387A-1KT; Sigma-Aldrich).

For collagen type II staining, antigens were enzymatically digested by incubation with 0.25% trypsin at 37°C for 10 minutes, then washed with PBS and further incubated with 1.45 U/mL hyaluronidase (Roth, Karlsruhe, Germany) at 37°C for 10 minutes. After washing in PBS, sections were further used for immunodetection with the specific antibody.

Cryosections (8 μm) were fixed for 10 minutes in acetone at –20°C and then air-dried. For immunodetection, after rehydration in PBS, unspecific binding sites were blocked with 10% normal goat serum (Dako Cytomation) for 1 hour at room temperature in a humidified atmosphere. Immunolocalization was performed, incubating sections with primary antibodies diluted in 2% bovine serum albumin/1% normal goat serum/PBS overnight at 4°C. The primary antibodies used were Rabbit α Collagen 1 (ab21286; Abcam, Cambridge, UK); Mouse α Collagen II (CP-18; Merck, Darmstadt, Germany); Rabbit α Ihh (ab39634; Abcam); Rat α Endomucin (HM1108; Hycult Biotech, Uden, the Netherlands); Rabbit α VEGF (sc-195; Santa Cruz Biotechnology, Heidelberg, Germany); Rabbit α Collagen 1 3/4 (0217-050; immunoGlobe, Himmelstadt, Germany); CD31 and α-SMA (from Dako Cytomation and Abcam, respectively); Keratin 14 (PRB-155P; Covance, Muenster, Germany); loricrin (PRB 145 PE; Covance); Rabbit anti-Ki67 (ab15580; Abcam), and Rabbit anti-MMP9 (ab38898; Abcam). After extensive washing, primary-bound antibodies were detected by using Alexa Fluor 488– or Alexa Fluor 594–conjugated secondary antibodies (Thermo Scientific) for 1 hour at room temperature. Nuclei were stained by using DAPI (1 μg/mL).

### Statistical Analysis

Statistical analyses were performed using GraphPad Prism 10.2.3 (GraphPad Software, San Diego, CA). Data normality was assessed by using the Shapiro-Wilk test before further analyses. Depending on whether data sets followed a Gaussian distribution, either parametric or nonparametric tests were applied, as indicated in the corresponding figure legends. The tests used for individual analysis and the number of biological replicates (n) are indicated in the figure legend for each figure.

## Results

### *Mmp13/Mmp14* DKO Mice Resemble *Mmp14*^*–/–*^ Mice in Size and Morphology

**Figure 1:**
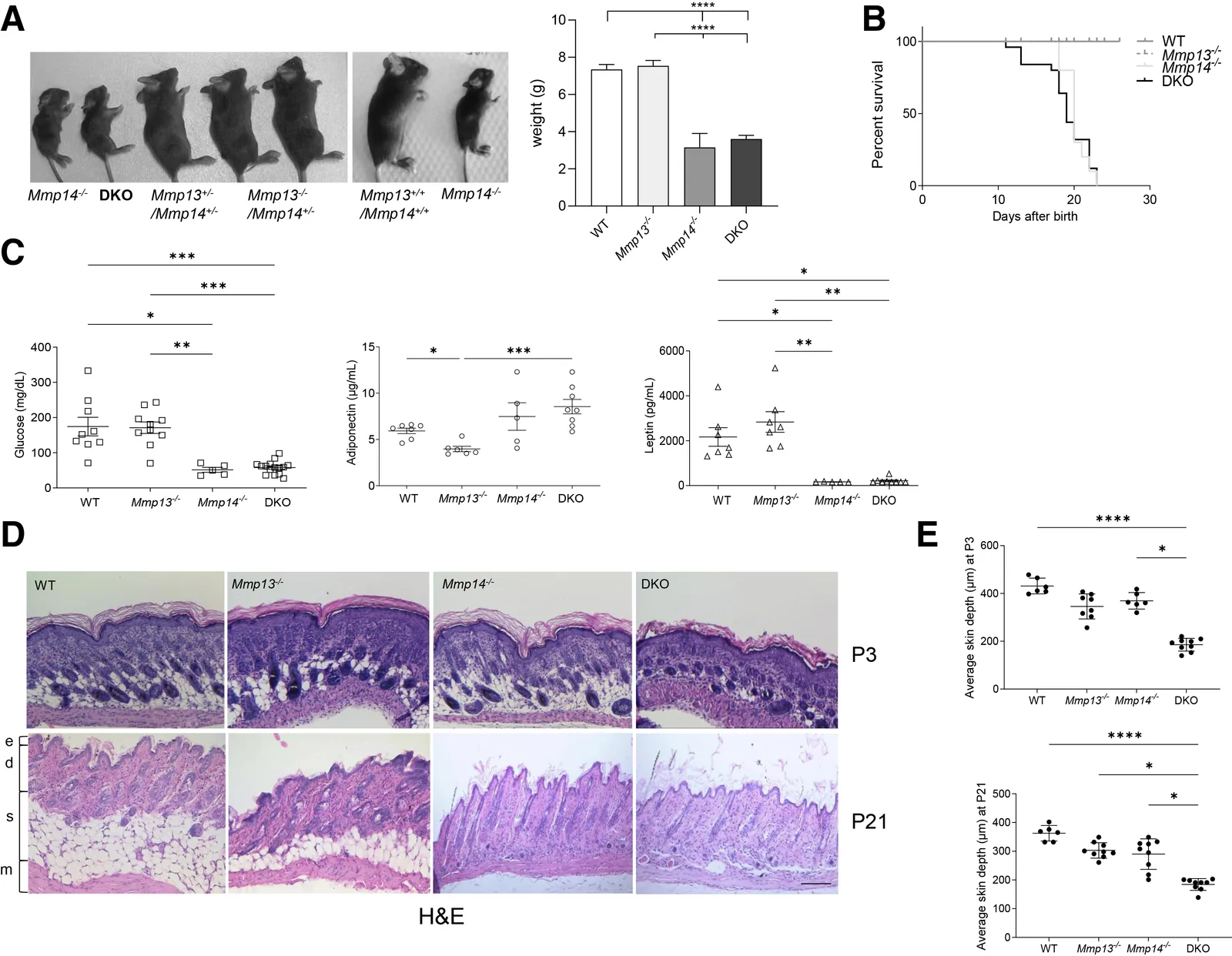
Phenotype of *Mmp13/Mmp14* double knockout (DKO) mice. **A:** The phenotypical appearance of *Mmp13*^*–/–*^ and *Mmp14*^*–/–*^ mice, heterozygous for one gene, and *Mmp13*^*–/–*^*/Mmp14*^*–/–*^ (DKO) mice at postnatal day 21 (P21) and their weight. **B:** Kaplan-Meier plot of survival in mice up to 3 weeks following birth. **C:** Blood glucose, adiponectin, and leptin levels in all mouse genotypes. Each dot represents data from a single mouse. **D:** Paraffin-embedded sections from the back skin of wild-type (WT), *Mmp13*^*–/–*^, *Mmp14*^*–/–*^, and DKO mice at postnatal day 3 (P3) and P21 were stained with hematoxylin and eosin (H&E) to compare overall skin structure. **E:** Depth of the skin from the upper epidermal layer to the underlying muscle was measured at P3 and P21, and the average is shown. Data are expressed as means ± SEM. WT, *n* = 29; *Mmp13*^*–/–*^, *n* = 29; *Mmp14*^*–/–*^, *n* = 9; DKO, *n* = 30 (**A**).∗*P* < 0.05, ∗∗*P* < 0.01, ∗∗∗*P* < 0.001, ∗∗∗∗*P* < 0.0001 (one-way analysis of variance). Scale bar = 100 μm (**D**). d, dermis; e, epidermis; f, fat; m, muscle.

To analyze the redundant and exclusive functions of MMP13 and MMP14 *in vivo*, mutants lacking both *Mmp13* and *Mmp14* were generated by crossing Mmp14^+/–^ and Mmp13^–/–^. The appearance of the mice with double deficiency, *Mmp13*^*–/–*^*/Mmp14*^*–/–*^ (DKO), recapitulates the phenotype of *Mmp14*^*–/–*^ (Figure 1A) with severe craniofacial dysmorphism, dwarfism, and weight loss, as previously described.^6^^,^^7^ Survival rates recorded from birth showed a survival trend in DKO offspring comparable to that of *Mmp14*^*–/–*^, with an average survival age of 21 days (Figure 1B). *Mmp13*^*–/–*^ had a normal lifespan, were fertile, and showed no visible phenotypes compared with WT mice (*Mmp13*^*+/+*^/*Mmp14*^*+/+*^).

As previously shown in *Mmp14*^*–/–*^ mice,^21^ significantly lower blood glucose levels were observed in *Mmp14*^*–/–*^ mice, which were comparably low in the DKO mice, compared with WT mice (Figure 1C). Levels of leptin in both mouse genotypes were reduced, whereas those of adiponectin were elevated (Figure 1C), resulting in substantial weight loss comparable to that reported earlier.^22^^,^^23^

**Figure 2:**
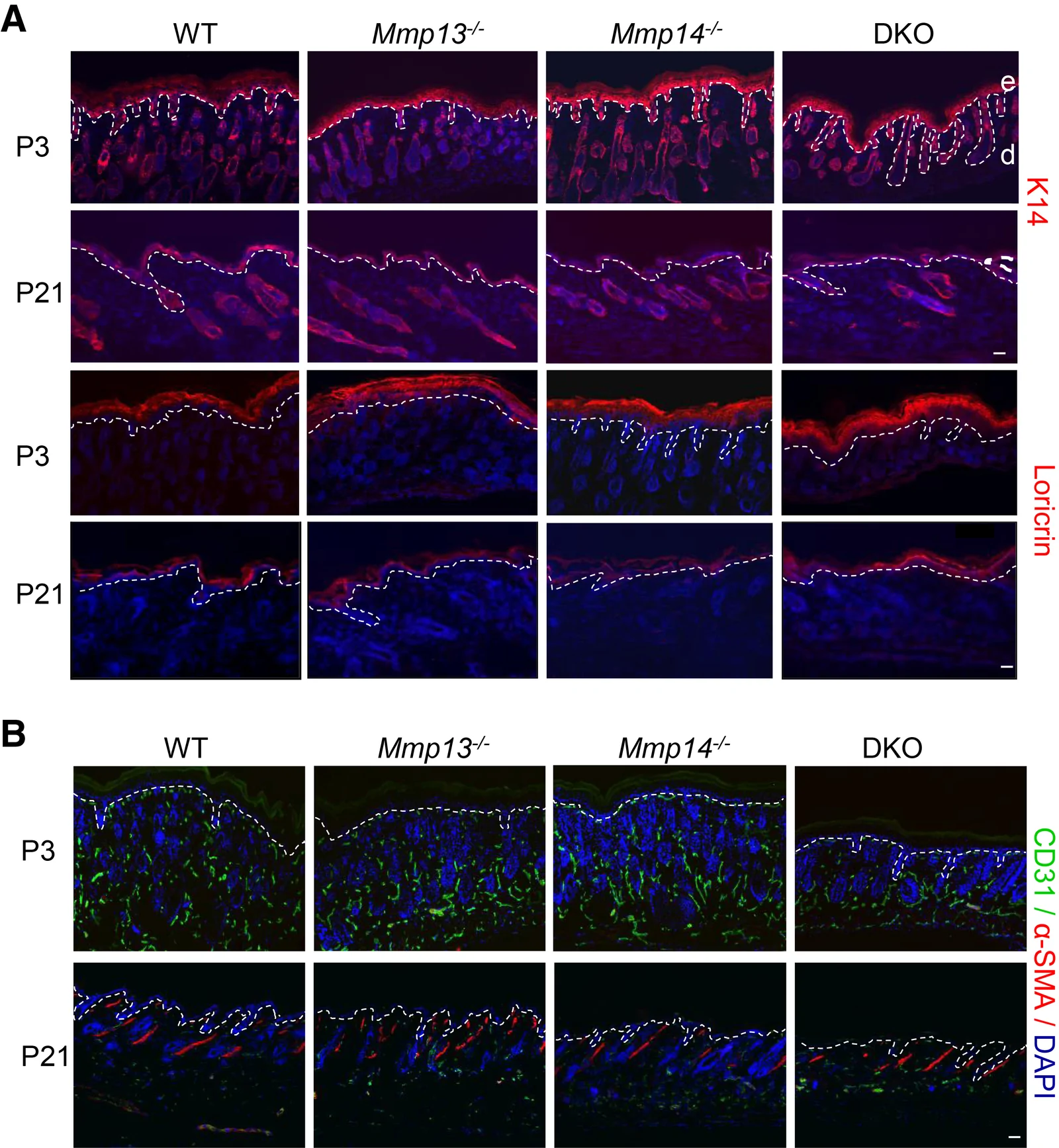
Early and late skin differentiation in the double knockout (DKO) mouse. **A:** Cryosections (8 μm) of wild-type (WT), single *Mmp13*^*–/–*^ and *Mmp14*^*–/–-*^ knockout, and DKO back skin at postnatal day 3 (P3) and postnatal day 21 (P21) were stained for K14 or loricrin (red). **B:** Alternatively, sections were immuno-stained using a specific antibody to CD31 (green), a marker for endothelial cells, and to α-smooth muscle actin (α-SMA) (red), a marker for myofibroblastic cells. A **white dotted line** marks the epidermal-dermal junction. DAPI blue staining identifies cell nuclei. Original magnification, ×100. Scale bars = 40 μm. d, dermis; e, epidermis.

Given the expectation that simultaneous loss of MMP13 and MMP14 would result in a pronounced skin phenotype, the general skin architecture of WT, *Mmp13*^*–/–*^, *Mmp14*^*–/–*^, and DKO mice was investigated at postnatal days 3 and 21 using hematoxylin and eosin staining of back skin (Figure 1D). Surprisingly, the skin of DKO mice closely resembled that of *Mmp14*^*–/–*^ animals, exhibiting reduced subcutaneous fat (Figure 1D) with an earlier onset of reduction in subcutaneous fat detected at postnatal day 3 and postnatal day 21 in the DKO specimen compared with the *Mmp14*^*–/–*^ mouse skin (Figure 1E) but no exacerbated or unique defects beyond those seen in the single knockout specimen. Despite the almost absent fat layer, no alterations in the distribution of markers of epidermal differentiation, including K14 and loricrin, and vascularization were indistinguishable across all genotypes, both shortly after birth (postnatal day 3) and at postnatal day 21 (Figure 2). These findings challenge the assumption of functional redundancy between MMP13 and MMP14 in skin development and suggest that neither protease is essential for epidermal or dermal morphogenesis and differentiation.

### Skeletal Phenotypes in DKO Mice

**Figure 3:**
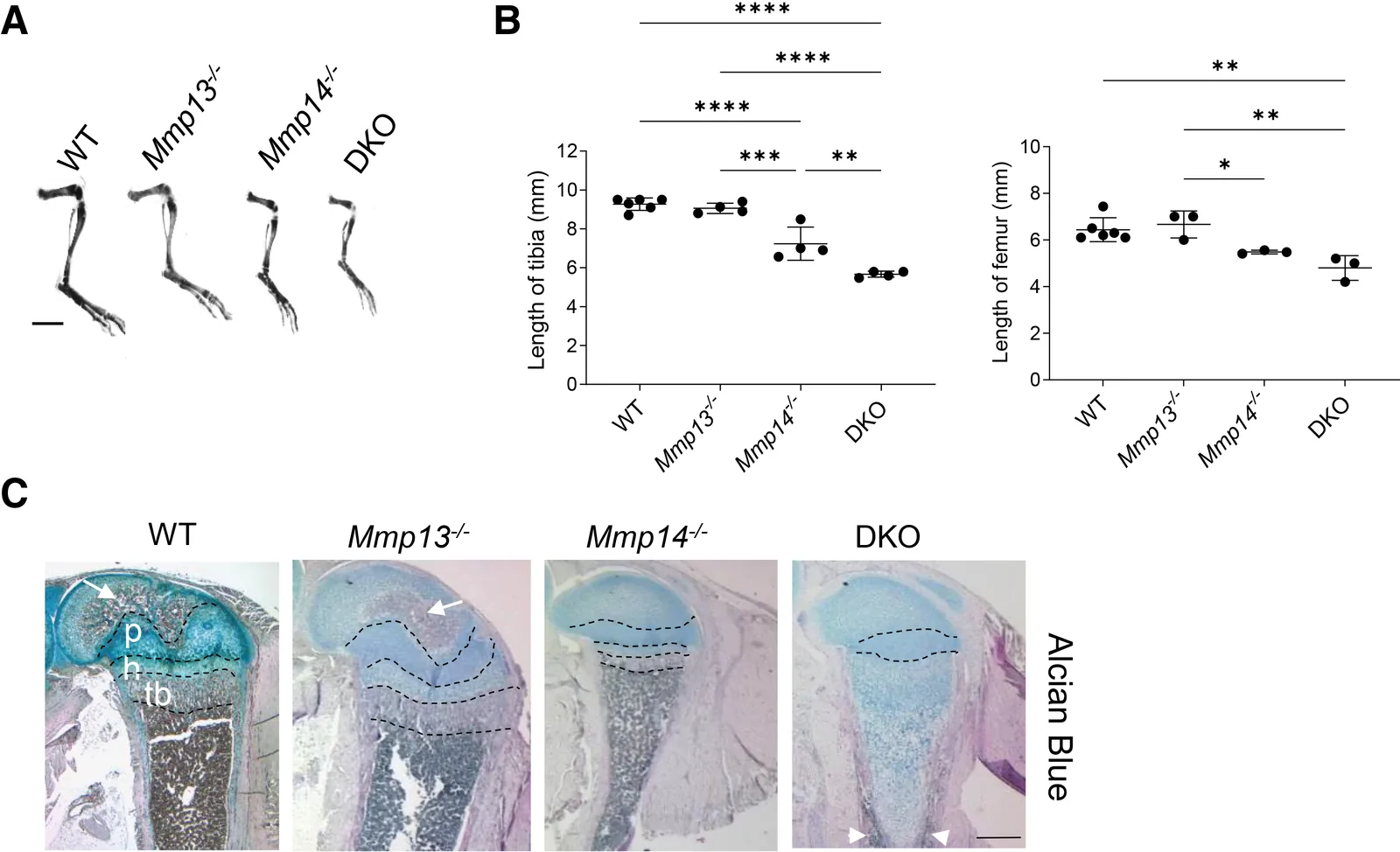
Double knockout (DKO) mice bone phenotype at postnatal day 14 (P14). **A:** Hind legs of all mouse genotypes at P14. Bone length in heterozygous animals does not significantly differ from their homozygous wild-type (WT) counterparts. **B:** Lengths of tibias and femurs were measured. The legs of *Mmp14*^*–/–*^ and DKO mice exhibit apparent shortening of the skeleton compared with other genotypes. **C:** Alcian Blue staining of femurs from P14 mice. The proliferative (p) zone, hypertrophic (h) zone, and area with trabecular bone (tb) are marked and outlined with **dashed lines**. The **white arrows** indicate the formed secondary ossification centers. No primary ossification center formation is observed (indicated by the **white arrowhead**) in the bones of DKO mice. Data are expressed as means ± SD. ∗*P* < 0.05, ∗∗*P* < 0.01, ∗∗∗*P* < 0.001, ∗∗∗∗*P* < 0.0001 (one-way analysis of variance). *n* = 5, each genotype (**B**). Scale bar = 500 μm (**C**).

Unlike skin, where the double deficiency does not cause a skin phenotype, in the skeletal system, a visible phenotype similar to that observed in *Mmp14*^*–/–*^ mice was present, including craniofacial dysmorphism and dwarfism. This aligns with the observed expression of both proteases during bone formation. When examining the bone development phenotypes observed earlier in *Mmp14*^*–/–*^ mice,^6^^,^^7^ membranous ossification in calvarial bones was also observed in DKO mice, resulting in incomplete sutures (data not shown). At P14, the tibia and femur were significantly shortened in *Mmp14*^*–/–*^ mice, with an even more pronounced shortening in DKO animals (Figure 3, A and B). This suggests that loss of both genes further disrupts skeletal growth, underscoring their additive effect in endochondral ossification–mediated bone growth.

**Figure 4:**
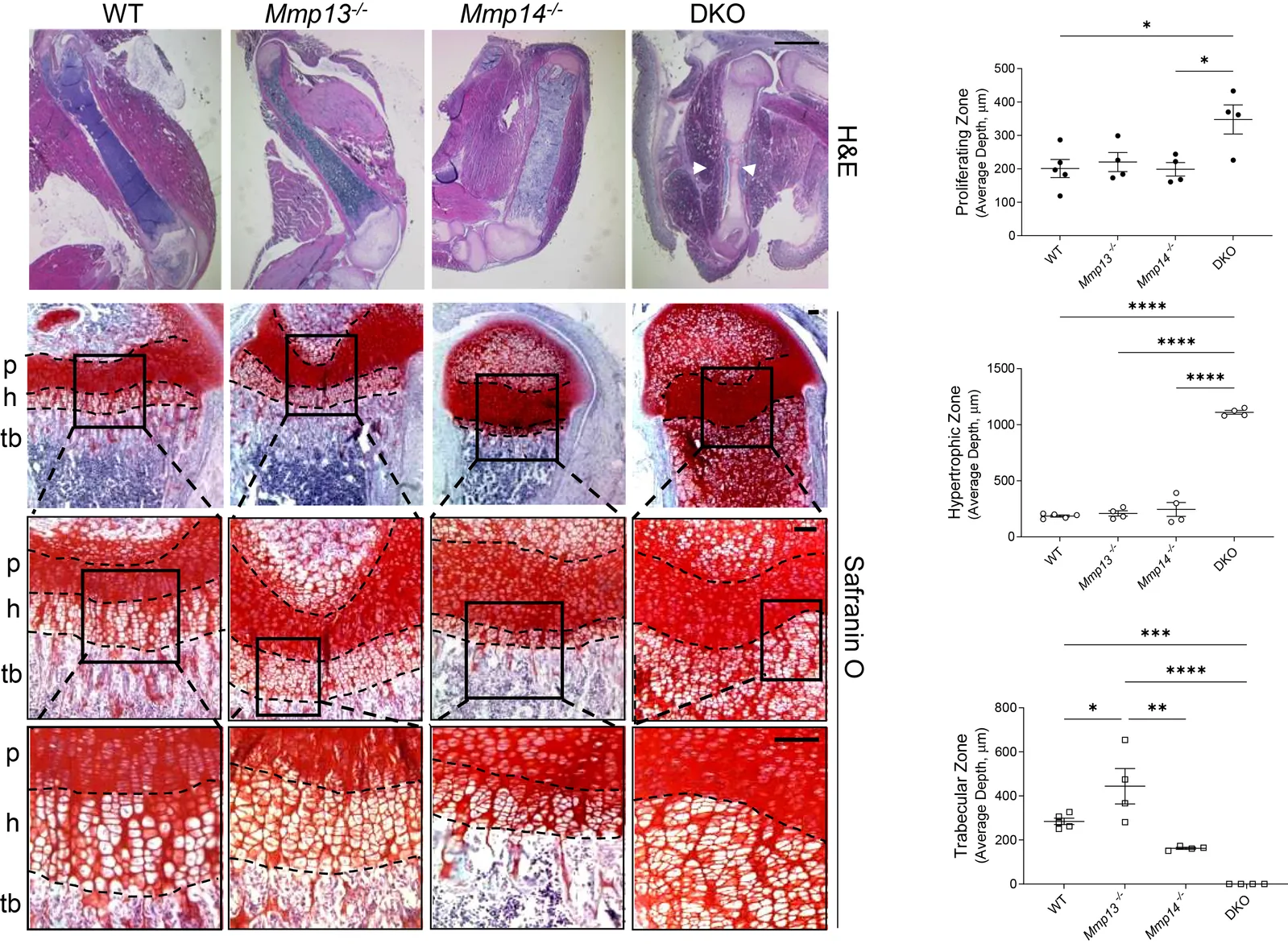
Safranin O staining of murine femurs at postnatal day 14. Legs of mice from all genotypes were fixed and embedded in paraffin. Sections of femurs (6 μm thick) were stained with Safranin O (red, cartilage’s proteoglycans), hematoxylin (purple, nuclei), and eosin (faint pink, cytoplasm) (H&E); different zones of the growth plate are marked by **dashed lines** (p = proliferative zone, h = hyperproliferative zone, tb = trabecular bone). No primary ossification center formation is observed (indicated by the **white arrowheads**) in the bones of DKO mice. All zones were measured, and the average depth of each is shown on the **right panel**. The area depicted in the box was further enlarged at magnifications of ×50, ×100, and ×200 (from top to bottom). ∗*P* < 0.05, ∗∗*P* < 0.01, ∗∗∗*P* < 0.001, ∗∗∗∗*P* < 0.0001 (one-way analysis of variance). Scale bars: 1000 μm (H&E); 100 μm (Safranin O).

When analyzing long bones at P14 with Alcian Blue, the WT, *Mmp13*^*–/–*^, and *Mmp14*^*–/–*^ long bones display a typically formed ossified channel within the diaphysis filled with bone marrow. In contrast, in DKO, the cartilage still extends through the diaphysis not being degraded, which is reminiscent of embryonal cartilaginous areas with the beginning of POC formation (Figures 3C and 4). A comparable histologic appearance was visible also in the tibias (Supplemental Figure S1A). Secondary ossification centers were formed in the WT and *Mmp13*^*–/–*^ epiphysis (Figure 3C) but not in the *Mmp14*^*–/–*^ and in the DKO bones at P14 (Figure 3C). Detection of proteoglycan-rich cartilage by Safranin O staining visualized the orderly organized growth plate cartilage, with a proliferation zone, hypertrophic chondrocytes, and trabecular bone (Figure 4) in WT and *Mmp13*^*–/–*^ mice, whereas the hypertrophic zone was thinner in *Mmp14*^*–/–*^. In contrast, a massive expansion was detected in DKO mice.

### POC Formation Is Delayed in DKO Mice

**Figure 5:**
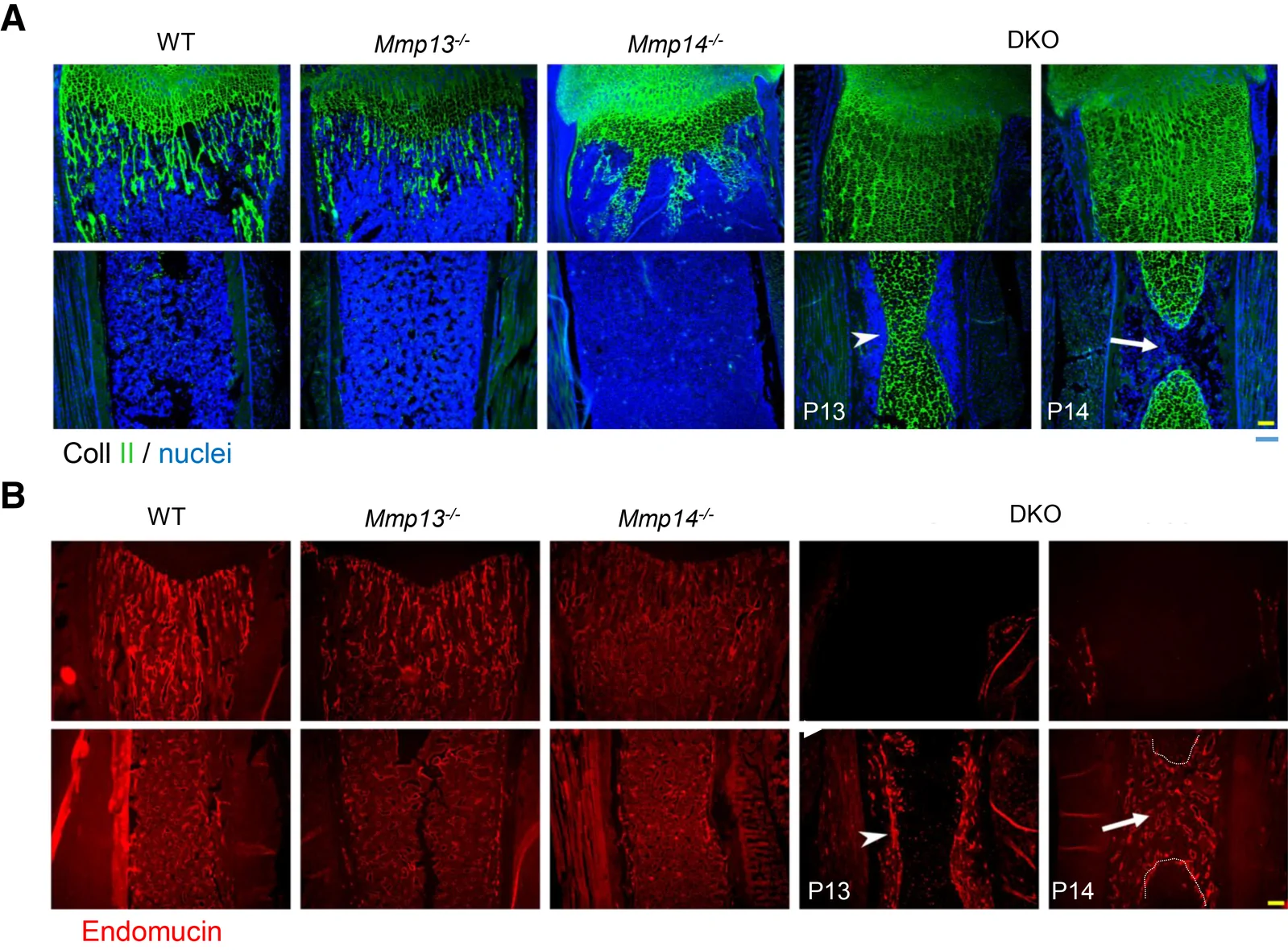
Localization of collagen type II (Coll II) and of vascular structures *in vivo*. **A:** Coll II (green) localization in sections (10 μm) of the decalcified murine femur from specimens of the four different genotypes [wild-type (WT), *Mmp13*^*–/–*^, *Mmp14*^*–/–*^, double knockout (DKO)] at postnatal day 14 (P14). DKO was analyzed at both postnatal day 13 (P13) (*n* = 5) and P14 (*n* = 5); representative images are shown. The **arrowhead** indicates Coll II expression in chondrocytes within the diaphysis of DKO at P13. At P14, with invasion, the central area is filled with bone marrow cells (**white arrow**). The metaphysis of the bones is shown in the **top row**, and the central part of the diaphysis is shown in the **bottom row**. **B:** Endomucin, a marker of endothelial cells, was analyzed in femurs at P14 (WT, *Mmp13*^*–/–*^, *Mmp14*^*–/–*^, and DKO; P13 and P14); representative pictures are shown. The **arrowhead** indicates that blood vessels have not yet invaded the center of the cartilage at P13 in DKO. In contrast, the **arrow** indicates a zone of vascular invasion at a later time point (P14), with the chondrocyte zone retracting (marked by a **dotted line**). The metaphysis of the bones is shown at the top, and the central part of the diaphysis is shown below. WT, *Mmp13*^*–/–*^, and *Mmp14*^*–/–*^ , each *n* = 5; DKO, *n* = 10 (**A** and **B**); P13 and P14, each *n* = 5. Scale bars = 100 μm (**A** and **B**).

Deposition of collagen type II, as the major fibrillar collagen in cartilage, is detected in proliferating and hypertrophic chondrocytes of developing bones, and its degradation is associated with the induction of differentiation of hypertrophic chondrocytes, vascular invasion, and formation of the POC.^24^ By immunofluorescence, in sections from bones at P14, collagen type II was detected in WT sections in branches of the spongiosa near the edge of the cartilaginous growth plates in WT and *Mmp13*^*–/–*^ (Figure 5A) sections. In *Mmp14*^*–/–*^ sections, collagen type II was detected in the cartilage of the growth plate with abnormal branches (Figure 5A). The latter is in line with previous investigations of *Mmp14*^*–/–*^ mice.^7^

In striking contrast, the diaphysis of the DKO femur was filled with collagen type II–positive chondrocytes at postnatal day 13 (P13) and P14; a central area was free of collagen type II, indicating that POC had started to form and that hypertrophic chondrocytes had begun degrading the collagenous matrix (Figure 5A). The formation of POC seen at P13 and P14 in DKO specimens (Figure 5A) was significantly delayed, as this process in WT specimens was described between embryonic day 13.5 (E13.5) and embryonic day 15.5.^25^

The time frame for POC formation was further underscored by an analysis of vascular structures within the postnatal bones using immunostaining for the sialoglycoprotein endomucin, a marker of endothelial cells.^26^ Vascular invasion from the perichondrium of the DKO cartilage started at P13 and thoroughly infiltrated the central diaphysis by P14 (Figure 5B). In all other analyzed mouse genotypes, at P14, bone growth and vascularization were further advanced, and all primary structures have been established. These data show the time point of POC formation in post-birth DKO bones between P13 and P14, thus indicating an extensive delay, but not complete impairment, in endochondral ossification. The delayed formation of POC in DKO mice is paralleled by reduced collagen type I processing and impaired vascular invasion.

A key factor orchestrating chondrocyte maturation, vascular invasion via vascular endothelial growth factor (VEGF) expression, and, ultimately, endochondral ossification is Ihh.^27^, ^28^, ^29^ Although often analyzed at the transcriptional level by *in situ* hybridization, Ihh protein expression and localization were analyzed in tissue using immunofluorescence. Ihh protein was detected in the metaphysis of the DKO bone and in extensions in the epiphysis of the same, which is comparable to the distribution observed in WT embryos (Supplemental Figure S1B) here and as shown previously.^30^ In the same areas of Ihh positivity, proliferation was also occurring, as indicated by the positivity to the Ki67 proliferation marker (Supplemental Figure S1B). These data suggest that chondrocyte differentiation has occurred. In addition, the expression of VEGFA, a general driver of vascular invasion in bone tissue during POC formation, is indirectly regulated by Ihh during bone formation.^31^^,^^32^

However, in developing DKO bones, VEGF was strongly expressed in the center of the collar at P13, comparable to WT bones at E13.5, and reduced at a later time point (postnatal day 19) (Supplemental Figure S2). Altogether, the expression patterns of collagens, invading vessels, Ihh, and VEGF indicate that the onset of endochondral bone formation in DKO is largely and strikingly delayed from E13.5/embryonic day 15.5 to P13/P14. The invasion of blood vessels into hypertrophic cartilage is coupled to the recruitment of chondroclastic/osteoclastic cells into the hypertrophic zone, with VEGF playing a role in both processes. Analysis of tartrate-resistant acid phosphatase–positive osteoclasts revealed positive cells lining trabecular surfaces in the metaphysis of WT, *Mmp13*^*–/–*^, and *Mmp14*^*–/–*^ bones where endochondral ossification normally proceeds (Supplemental Figure S3). In contrast, DKO bones lacked tartrate-resistant acid phosphatase–positive cells in the metaphysis, and osteoclasts localized on the lateral side of the hypertrophic cartilage, likely guided there by sidewise-penetrating blood vessels (Supplemental Figure S3). In addition, in these areas, comparable to embryos at E13.5, MMP9-positive cells were detected in the perichondrium and in the cavity, likely corresponding to osteoclasts/chondroclasts, as previously shown.^33^

These findings indicate that removing both MMPs does not significantly impair chondrocyte differentiation and chondroclastic/osteoclastic cells recruitment, although it cannot be entirely ruled out that a minor role of chondrocyte differentiation in delaying POC formation.

**Figure 6:**
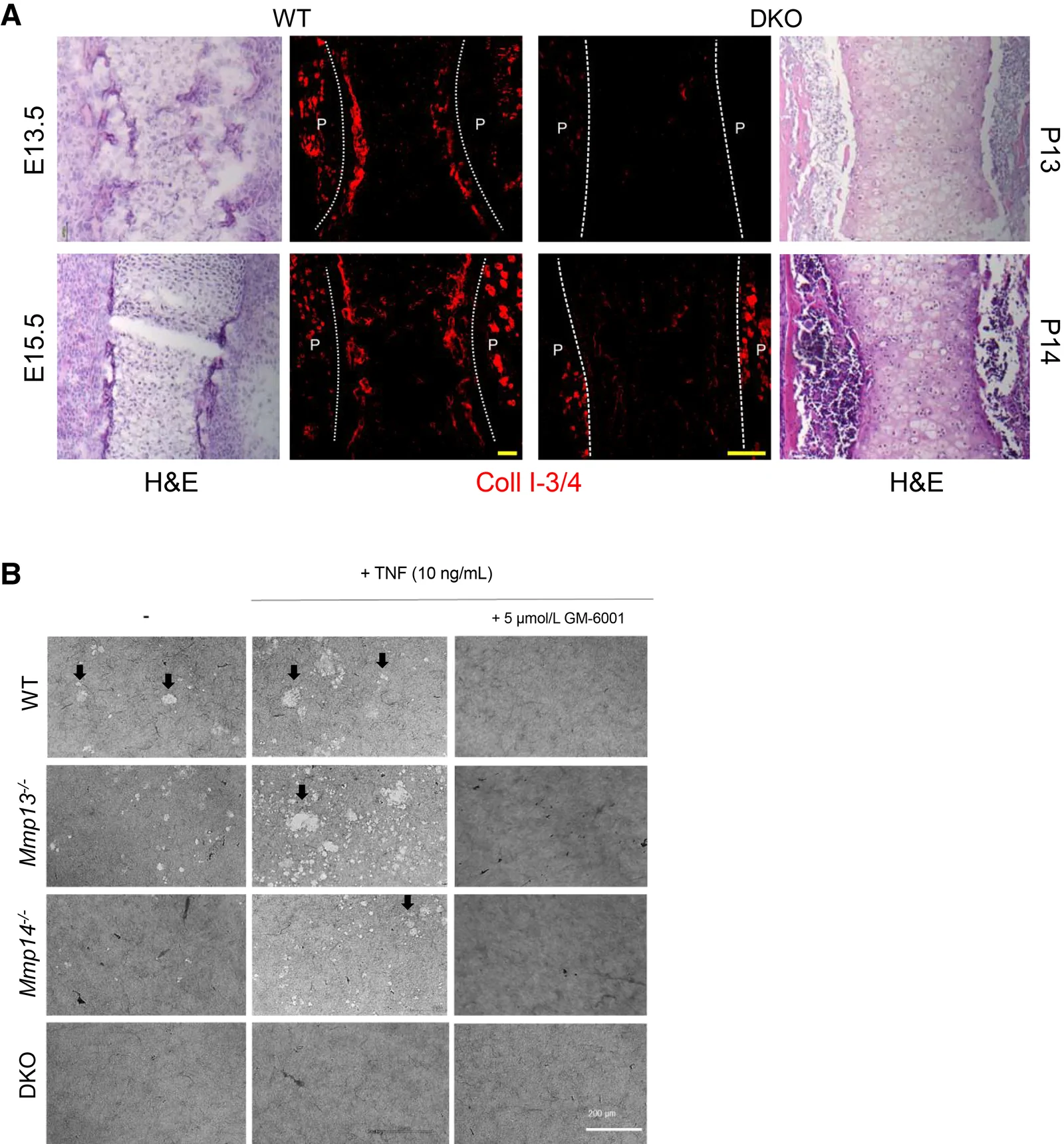
Collagen type I degradation *in vivo* and *in vitro*. **A:** Collagen type I 3/4 (Coll I-3/4) neoepitope immunolocalized (red) in sections of the native murine femur. Images are representative of wild-type specimens stained at embryonic day 13.5 (E13.5) and embryonic day 15.5 (E15.5), and of double knockout (DKO) specimens at postnatal day 13 (P13) and postnatal day (P14). The diaphysis of both genotypes is shown, with **broken lines** parallel to the perichondrium (P). **B:** Collagenolytic activity of fibroblasts from all genotypes seeded on a reconstituted type I collagen film in the absence (–) or presence of tumor necrosis factor (TNF) or GM6001 inhibitor. Collagen degradation appears as white areas (indicated by **black arrows**) on a blue background (shown in darker gray). The experiment was repeated twice with duplicates. E13.5 and E15.5, each *n* = 2; P13 and P14, each *n* = 5 (**A**). Scale bars: 100 μm (**A**); 200 μm (**B**). H&E, hematoxylin and eosin.

**Supplemental Figure S4:**
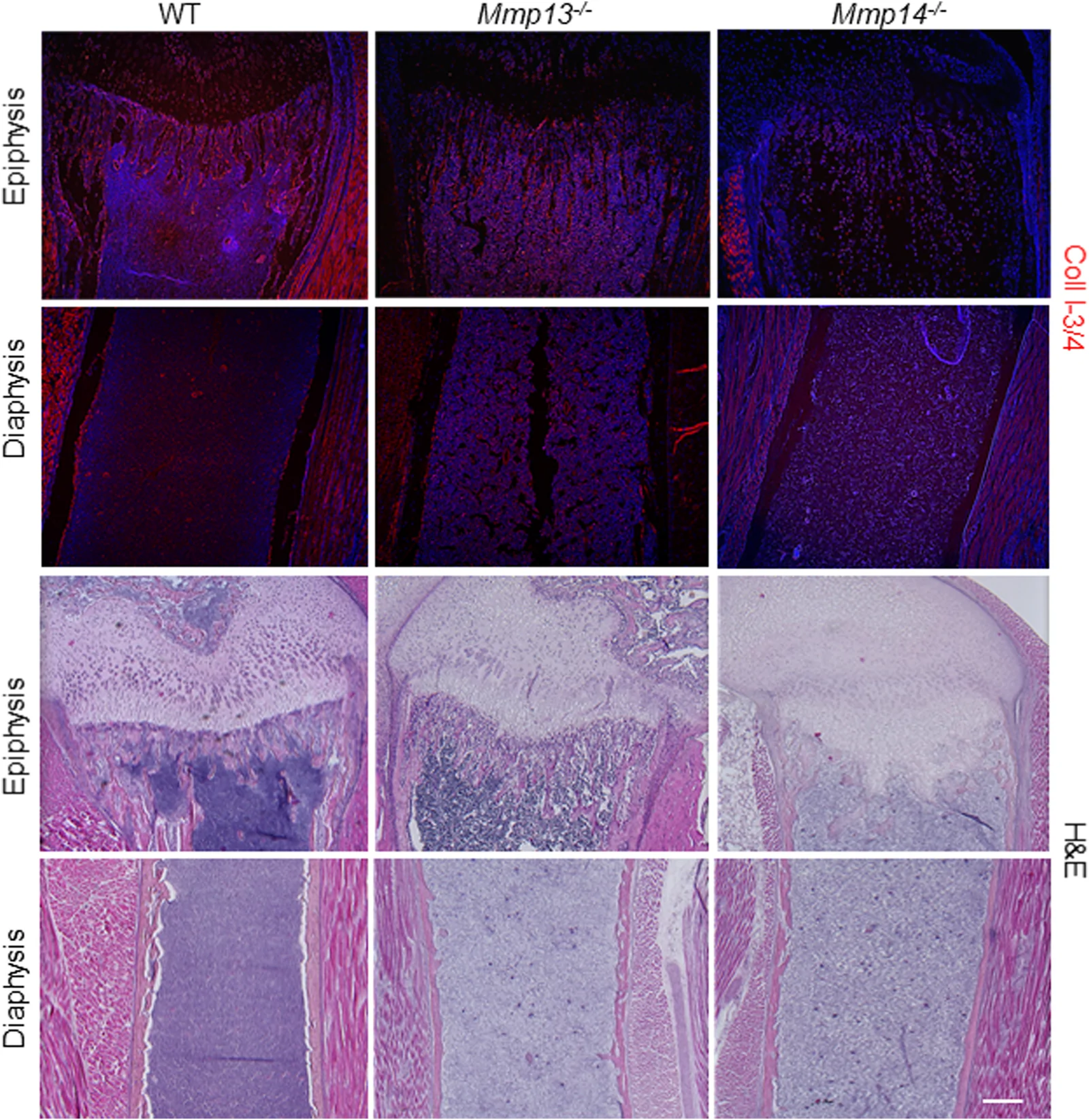
Collagen type I degradation *in vivo*. Collagen type I 3/4 (Coll I-3/4) neoepitope immunolocalized (red) in sections of the native murine femur. Images are representative of wild-type (WT), Mmp13^–/–^, and Mmp14^–/–^ bone specimens stained at postnatal day 14 and double knockout specimens at postnatal day 13. The epiphysis (upper) and diaphysis (lower) are shown. Scale bar = 100 μm. H&E, hematoxylin and eosin.

Collagen type I deposition in the bone collar around the cartilage anlage, which is also rich in collagen type II, has been previously reported before the initiation of POC formation.^34^ Therefore, vascular invasion of the cartilage anlage requires the presence of collagenolytic activities. Both MMP13 and MMP14 are collagenases that degrade collagen type I.^2^ To determine whether impaired collagenolysis caused the significant delay in vascular invasion when crossing the collagen type I belt, an antibody recognizing the 3/4 neoepitope of collagen type I, resulting from cleavage, was used to assess proteolytic processing in the tissue.^35^ Collagen type I degradation products were mainly detected in the perichondrium in WT mice at E13.5 (Figure 6A) and within the diaphyseal center at embryonic day 15.5, with sizable, remodeled collagen type I in the surrounding soft tissues. Minimal, if any, processed collagen type I was detected in DKO mice at P13, but more was observed at P14 (Figure 6A). Thus, although collagen type I processing in DKO mice appears impaired up to P13, this defect is partially compensated by P14, when collagen type I processing facilitates vascular invasion of the tissue. At P14 in WT, *Mmp13*^*–/–*^, and *Mmp14*^*–/–*^ mice, areas of collagen degradation (as from collagen type I 3/4 detection) are detected in the epiphyseal regions in correspondence with the trabecular bone where remodeling is occurring; in the diaphyseal regions, however, almost no collagen degradation is found (Supplemental Figure S4). These data indicate that collagen degradation remains lower after POC formation during postnatal stages.

Using an *in vitro* degradation assay^8^ and fibroblasts isolated from the back skin of all mouse genotypes, areas of fibrillar collagen degradation were detected by fibroblasts from WT and *Mmp13*^*–/–*^ mice cultured on that substrate. The presence of tumor necrosis factor enhanced this effect, and this effect was blocked by the MMP inhibitor GM6001 (Figure 6B).

**Supplemental Figure S5:**
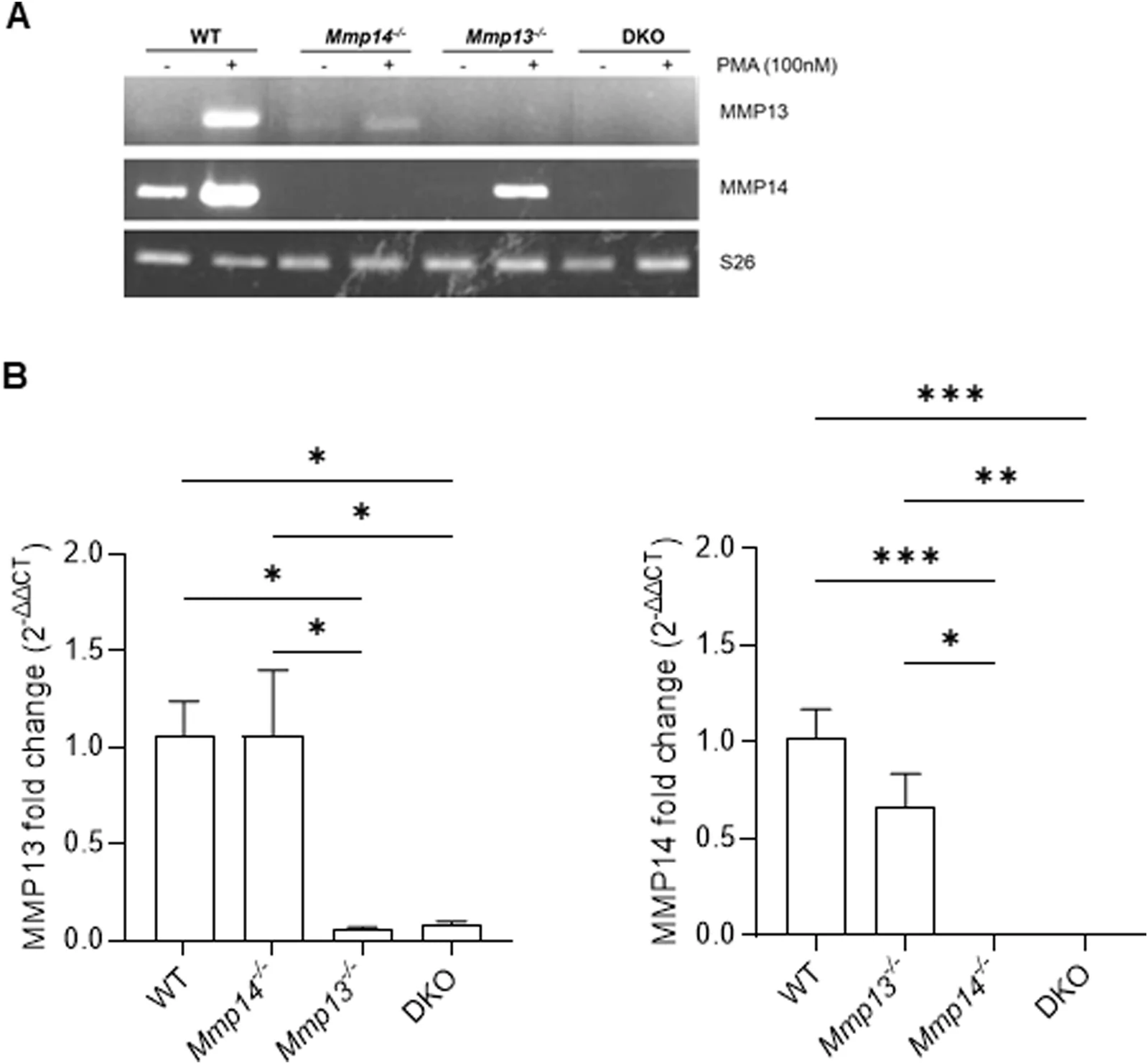
MMP13 and MMP14 expression in stimulated fibroblasts and skin. **A** and **B:** RNA was derived from untreated (–) and phorbol 12-myristate 13-acetate (PMA)-treated (100 nmol/L) (+) murine fibroblasts from all genotypes after 24 hours (**A**) or by punches from the back skin of the different mice genotypes (**B**), reverse transcribed, and cDNA was amplified to detect levels of MMP13 and MMP14 by RT-PCR or real-time quantitative PCR . The S26 levels served as a reference transcript. ∗*P* ˂ 0.05, ∗∗*P* ˂ 0.01, ∗∗∗*P* ˂ 0.001, one-way analysis of variance. DKO, double knockout; WT, wild type.

However, *Mmp14*^*–/–*^ fibroblasts under standard culture conditions did not degrade fibrillar collagen, and stimulation with tumor necrosis factor led to only minor degradation. Both the slightly enhanced collagen degradation in *Mmp13*^*–/–*^ and the minor degradation in *Mmp14*^*–/–*^ fibroblasts may result from reciprocal activity. Indeed, i*n vitro*, as observed in PMA-stimulated fibroblasts, both proteases were expressed when reciprocally deleted (Supplemental Figure S5A). PMA was used to stimulate MMP13 expression that would be otherwise almost undetectable in these conditions. Consistent with these data is the expression of both transcripts in skin *in vivo* (Supplemental Figure S5B); thus, because the deletion of both collagenases in fibroblasts resulted in an almost complete loss of collagen-degrading ability (Figure 6B), they may compensate for each other in this activity.

## Discussion

Tissue remodeling by MMPs is fundamental not only to pathologic processes such as tumor growth but also to the intricate processes of organ development. Despite extensive characterization of MMP-mediated ECM turnover, significant gaps remain in the understanding of how specific MMPs orchestrate collagen remodeling that underpins tissue integrity and morphogenesis. Among ECM proteins, collagens [particularly interstitial collagens (types I to III)] are the principal substrates for several MMPs, including MMP1, MMP2, MMP8, MMP13, MMP14 (membrane type 1 MMP), MMP18, and MMP22.^2^ Notably, MMP14 stands out for its nonredundant role in postnatal development, as systemic deletion of this enzyme results in early lethality and profound defects in bone and soft tissue formation, highlighting the enzyme’s indispensable role in mammalian development.^6^^,^^7^^,^^36^ Although MMP13 and MMP14 display substrate preferences for collagen types II and I, respectively,^37^ both possess the capacity to cleave fibrillar collagens,^2^ suggesting both overlapping and distinct biological functions. Furthermore, evidence of compensatory mechanisms, such as the moderation of trabecular bone volume in *Mmp13*-deficient mice via MMP14 activity, illustrates a dynamic interplay between these enzymes *in vivo*.^19^

Given these insights, the current study aimed to address critical gaps in understanding how the substrate specificity and compensatory dynamics of MMP13 and MMP14 govern ECM remodeling during development and disease in two collagen-rich tissues: skin and skeletal system. Notably, the findings revealed that a combined deficiency of both collagenases, *Mmp13* and *Mmp14*, does not result in early postnatal skin fibrosis, suggesting that these enzymes function in a tissue- or time-specific manner during dermal collagen remodeling. This observation points to additional proteolytic or non-proteolytic mechanisms, potentially involving other MMPs or regulatory pathways, that maintain ECM integrity during early life. However, loss of both collagenases leads to earlier subcutaneous fat loss, indicating that MMP13 plays a role in the early stages of fat formation. In contrast, loss of *Mmp14*, as previously shown,^38^ causes fat loss, but that was detected here at a later time point from birth. Although this is an intriguing finding, more research is needed to understand the molecular mechanisms by which both collagenases contribute to the development of subcutaneous fat tissue. The absence of a major skin phenotype in double-deficient mice in the early postnatal period suggests that the critical functions of MMP13 and MMP14 in skin may emerge later, as shown for MMP14 in adult skin, or under conditions of stress such as wound healing or fibrosis.^5^^,^^8^ Alternatively, other proteases or non-enzymatic mechanisms may compensate, further highlighting the complexity of ECM regulation.

The combined deficiency of *Mmp13* and *Mmp14* closely resembles several features of *Mmp14* deficiency alone, including early death. This overlap highlights the crucial role of MMP14, as seen in previous models lacking this enzyme, and is further supported by lower blood glucose and leptin levels. In addition, the increase in adiponectin, a hormone involved in triglyceride regulation,^23^ aligns with the higher adiponectin levels reported in *Mmp14*^*–/–*^ mice.^21^ These metabolic issues, consistent with findings in *Mmp14*^*–/–*^ mice,^21^ strongly suggest that malnutrition is a primary factor contributing to early death in DKO animals.

However, in DKO animals, there are additional severe disruptions in bone development and unique phenotypes, such as shortened tibias and femurs. The bone phenotypes seen in the DKO animal emphasize the importance of both proteases in endochondral ossification and suggest that their combined absence worsens bone development more than in single knockouts. The key phenotype in DKO mice is a notable delay in POC formation, along with a significantly enlarged hypertrophic cartilage zone compared with single knockouts. As a result, both the tibia and femur are shorter in the absence of both collagenases. This defect had not been previously observed in the *Mmp14*^*–/–*^ mice^6^^,^^7^ or during the embryonic development of *Mmp13*^*–/–*^ mice.^15^^,^^19^

Delayed vascular invasion due to impaired collagen remodeling/removal by collagenases suggests a compensatory role for MMP13 and MMP14 in vascular invasion, which underpins POC formation. The DKO model exhibited delayed vascular invasion through the cartilage anlage cuff, accompanied by impaired collagen type I degradation before P13. The lack of these collagenases may slow penetration through the bone collar’s collagen type I, thereby limiting radial expansion and scaffold formation. In agreement, defects in collagen breakdown were confirmed *in vitro* using fibrillar collagen degradation assays and fibroblasts from single knockout and DKO mice. Therefore, although it has been shown that, *in vitro*, MMP14 (not MMP13) is necessary for migration or sprouting in collagen type I matrices,^39^ *in vivo*, this process may also be supported by MMP13 to compensate for the loss of MMP14.

Although MMP2 and MMP9 do not significantly contribute to interstitial collagen turnover,^38^ this does not exclude the possibility that they may exert collagenolytic activity *in vivo* to compensate for the deficiency of both collagenases. Indeed, although there is ambiguity regarding the collagenolytic activity of MMP2 and MMP9, several reports have shown that both can cleave collagens *in vitro*.^40^

However, in *Mmp2/14* DKO mice, which die soon after birth, but not in single *Mmp2*^*–/–*^ mice, changes in growth plate vascularization and ossification occur. Nevertheless, POCs form and a notably shortened ossification zone develops.^41^ However, no assessments of collagen type I or II degradation were made in that study. In contrast, enlarged hypertrophic cartilage is observed in *Mmp9* and *Mmp9/13* knockouts, with the latter also exhibiting delayed formation of POCs.^19^^,^^42^ Although these findings suggest overlapping defects in cartilage remodeling, direct comparison with the DKO mouse phenotype is limited by differences in the developmental stages examined.

The *Mmp9/13* knockout mouse phenotype was linked to reduced degradation of collagen type II during vascular canal invasion, which, in the transformation of the epiphysis anlage in WT mice, is mainly processed by MMP13. However, a minor role for MMP14 in vascular invasion of the canal-blind end cannot be ruled out.^43^ In contrast, in *Mmp13* and *Mmp14* DKO mice, the observed alteration in collagen type I breakdown may delay POC formation by impairing the invasion of the collagen type I–rich bone collar during embryonic stages. This delay is eventually resolved after P13, allowing vascular invasion into the anlage and resulting in complete central bone vascularization. However, the mechanisms of this later-stage collagen breakdown, as well as the potential compensatory pathways and independent effects, remain unknown. In addition, a limitation of this study is that the deficiency of *Mmp13* and *Mmp14* is not tissue specific, which prevents distinguishing the primary cell–specific effects. In addition, potential sex-specific effects could not be excluded because mixed sex litters were used.

In conclusion, the persistence of hypertrophic cartilage and delayed vascular invasion in DKO mice highlights the cooperative functions of MMP13 and MMP14 in regulating key steps of endochondral ossification. The data show that these proteases contribute both uniquely and redundantly to tissue development, particularly in bone remodeling. The more severe phenotypes observed in DKOs compared with single mutants underscore the complexity of MMP-mediated processes and highlight the need for further investigation into compensatory interactions and broader physiological roles.

## Disclosure Statement

None declared.

## Supplemental Data

Supplemental Figure S1 Morphology of the tibia. **A:** Sections of tibia (6 μm thick) were stained with hematoxylin (purple, nuclei), and eosin (faint pink, cytoplasm) [hematoxylin and eosin (H&E)]. **B:** Ihh (red) and Ki67 (green) are immuno-localized in sections of the native murine femur. Representative bone tissue specimen of the wild- type (WT) section stained at embryonic day 13.5 (E13.5) as well as of the double knockout (DKO) section at postnatal day 14 (P14). The middle picture shows the bone stained by H&E. A **dashed line** indicates the areas magnified on the sides. The diaphyseal center and the upper epiphyses are shown. The **white arrows** indicate areas of high Ihh expression, where prominent staining with the proliferation marker Ki67 is also detected, in the immunofluorescence image, and **black arrows** indicate the corresponding area in the histologic H&E–stained section. E13.5, *n* = 5; DKO, *n* = 4 (**B**). Original magnification: ×25 (H&E); ×100 (Ihh). Scale bars: 1000 μm (**A**); 200 μm (**B**).

Supplemental Figure S2 Vascular endothelial growth factor immuno-localization (red) in sections of the native murine femur. Representative bone tissue specimen of the wild-type (WT) stained at embryonic day 13.5 (E13.5) and the double knockout (DKO) at postnatal day 14 (P14) and postnatal day 19 (*n* = 2). The diaphysis (DP) is indicated by a **dashed line**. E13.5, *n* = 5; P14, *n* = 4; P19, *n* = 2. MP, metaphysis. Original magnification, ×100. Scale bar = 200 μm.

Supplemental Figure S3 **A:** Tartrate-resistant acid phosphatase (TRAP) staining in sections (10 μm) of the decalcified murine femur. Images are representative of wild-type (WT), Mmp13^–/–^, and Mmp14^–/–^ specimens stained at postnatal day 14 (P14), and double knockout (DKO) specimens at postnatal day 13 (P13). The epiphysis of the bones is shown in the **top row**, and the central part of the diaphysis, including the bone wall, is shown in the **bottom row**. TRAP-positive cells are stained purple and marked with **arrowheads**; magnified areas of specific staining are shown in the insets. Cell nuclei are visible in green color. **B:** Representative bone tissue specimen of the WT specimen stained for MMP9 (in red) at embryonic day 13.5 (E13.5) and of the DKO specimen at P14. Positive staining (**white arrows**) is detected in cells of the bone collar and partly within the forming bone cavity. P14 specimens, *n* = 5; P13 specimens, *n* = 4 (**A**). Scale bars: 100 μm (**A**); 200 μm (**B**).

## References

[1] Gelse K., Pöschl E., Aigner T. (2003). Collagens—structure, function, and biosynthesis. Adv Drug Deliv Rev.

[2] Fields G.B. (2013). Interstitial collagen catabolism. J Biol Chem.

[3] Sprangers S., Everts V. (2019). Molecular pathways of cell-mediated degradation of fibrillar collagen. Matrix Biol.

[4] Hartenstein B., Dittrich B.T., Stickens D., Heyer B., Vu T.H., Teurich S., Schorpp-Kistner M., Werb Z., Angel P. (2006). Epidermal development and wound healing in matrix metalloproteinase 13-deficient mice. J Invest Dermatol.

[5] Hattori N., Mochizuki S., Kishi K., Nakajima T., Takaishi H., D’Armiento J., Okada Y. (2009). MMP-13 plays a role in keratinocyte migration, angiogenesis, and contraction in mouse skin wound healing. Am J Pathol.

[6] Zhou Z., Apte S.S., Soininen R., Cao R., Baaklini G.Y., Rauser R.W., Wang J., Cao Y., Tryggvason K. (2000). Impaired endochondral ossification and angiogenesis in mice deficient in membrane-type matrix metalloproteinase I. Proc Natl Acad Sci U S A.

[7] Holmbeck K., Bianco P., Caterina J., Yamada S., Kromer M., Kuznetsov S.A., Mankani M., Robey P.G., Poole A.R., Pidoux I., Ward J.M., Birkedal-Hansen H. (1999). MT1-MMP-deficient mice develop dwarfism, osteopenia, arthritis, and connective tissue disease due to inadequate collagen turnover. Cell.

[8] Zigrino P., Brinckmann J., Niehoff A., Lu Y., Giebeler N., Eckes B., Kadler K.E., Mauch C. (2016). Fibroblast-derived MMP-14 regulates collagen homeostasis in adult skin. J Invest Dermatol.

[9] Kümper M., Zamek J., Steinkamp J., Pach E., Mauch C., Zigrino P. (2022). Role of MMP3 and fibroblast-MMP14 in skin homeostasis and repair. Eur J Cell Biol.

[10] Krane S.M., Inada M. (2008). Matrix metalloproteinases and bone. Bone.

[11] Takahashi M., Fujikawa K., Angammana R., Shibata S. (2019). An in situ hybridization study of MMP-2, -9, -13, -14, TIMP-1, and -2 mRNA in fetal mouse mandibular condylar cartilage as compared with limb bud cartilage. Gene Expr Patterns.

[12] Munoz-Saez E., Moracho N., Learte A.I.R., Arroyo A.G., Sánchez-Camacho C. (2021). Dynamic expression of membrane type 1-matrix metalloproteinase (Mt1-mmp/Mmp14) in the mouse embryo. Cells.

[13] Chu T.L., Chen P., Yu A.X., Kong M., Tan Z., Tsang K.Y., Zhou Z., Cheah K.S.E. (2023). MMP14 cleaves PTH1R in the chondrocyte-derived osteoblast lineage, curbing signaling intensity for proper bone anabolism. Elife.

[14] Tuckermann J.P., Pittois K., Partridge N.C., Merregaert J., Angel P. (2000). Collagenase-3 (MMP-13) and integral membrane protein 2a (Itm2a) are marker genes of chondrogenic/osteoblastic cells in bone formation: sequential temporal, and spatial expression of Itm2a, alkaline phosphatase, MMP-13, and osteocalcin in the mouse. J Bone Miner Res.

[15] Inada M., Wang Y., Byrne M.H., Rahman M.U., Miyaura C., López-Otín C., Krane S.M. (2004). Critical roles for collagenase-3 (Mmp13) in development of growth plate cartilage and in endochondral ossification. Proc Natl Acad Sci U S A.

[16] Evans B.R., Mosig R.A., Lobl M., Martignetti C.R., Camacho C., Grum-Tokars V., Glucksman M.J., Martignetti J.A. (2012). Mutation of membrane type-1 metalloproteinase, MT1-MMP, causes the multicentric osteolysis and arthritis disease Winchester syndrome. Am J Hum Genet.

[17] Winchester P., Grossman H., Lim W.N., Danes B.S. (1969). A new acid mucopolysaccharidosis with skeletal deformities simulating rheumatoid arthritis. Am J Roentgenol Radium Ther Nucl Med.

[18] Cohen A.H., Hollister D.W., Reed W.B. (1975). The skin in the Winchester syndrome. Arch Dermatol.

[19] Stickens D., Behonick D.J., Ortega N., Heyer B., Hartenstein B., Yu Y., Fosang A.J., Schorpp-Kistner M., Angel P., Werb Z. (2004). Altered endochondral bone development in matrix metalloproteinase 13-deficient mice. Development.

[20] Kennedy A.M., Inada M., Krane S.M., Christie P.T., Harding B., López-Otín C., Sánchez L.M., Pannett A.A.J., Dearlove A., Hartley C., Byrne M.H., Reed A.A.C., Nesbit M.A., Whyte M.P., Thakker R.V. (2005). MMP13 mutation causes spondyloepimetaphyseal dysplasia, Missouri type (SEMD(MO). J Clin Invest.

[21] Mori H., Bhat R., Bruni-Cardoso A., Chen E.I., Jorgens D.M., Coutinho K., Louie K., Bowen B.B., Inman J.L., Tecca V., Lee S.J., Becker-Weimann S., Northen T., Seiki M., Borowsky A.D., Auer M., Bissell M.J. (2016). New insight into the role of MMP14 in metabolic balance. PeerJ.

[22] D’Souza A.M., Neumann U.H., Glavas M.M., Kieffer T.J. (2017). The glucoregulatory actions of leptin. Mol Metab.

[23] Yanai H., Yoshida H. (2019). Beneficial effects of adiponectin on glucose and lipid metabolism and atherosclerotic progression: mechanisms and perspectives. Int J Mol Sci.

[24] Kozhemyakina E., Lassar A.B., Zelzer E. (2015). A pathway to bone: signaling molecules and transcription factors involved in chondrocyte development and maturation. Development.

[25] Langen U.H., Pitulescu M.E., Kim J.M., Enriquez-Gasca R., Sivaraj K.K., Kusumbe A.P., Singh A., Di Russo J., Bixel M.G., Zhou B., Sorokin L., Vaquerizas J.M., Adams R.H. (2017). Cell-matrix signals specify bone endothelial cells during developmental osteogenesis. Nat Cell Biol.

[26] Sivaraj K.K., Adams R.H. (2016). Blood vessel formation and function in bone. Development.

[27] Karsenty G., Wagner E.F. (2002). Reaching a genetic and molecular understanding of skeletal development. Dev Cell.

[28] Goldring M.B., Tsuchimochi K., Ijiri K. (2006). The control of chondrogenesis. J Cell Biochem.

[29] Mackie E.J., Tatarczuch L., Mirams M. (2011). The skeleton: a multi-functional complex organ: the growth plate chondrocyte and endochondral ossification. J Endocrinol.

[30] Hattori T., Müller C., Gebhard S., Bauer E., Pausch F., Schlund B., Bösl M.R., Hess A., Surmann-Schmitt C., von der Mark H., de Crombrugghe B., von der Mark K. (2010). SOX9 is a major negative regulator of cartilage vascularization, bone marrow formation and endochondral ossification. Development.

[31] Yang Y.-Q., Tan Y.-Y., Wong R., Wenden A., Zhang L.-K., Rabie A.B.M. (2012). The role of vascular endothelial growth factor in ossification. Int J Oral Sci.

[32] Zhang C., Zhao R., Dong Z., Liu Y., Liu M., Li H., Yin Y., Che X., Wu G., Li G., Li P., Wei X., Yang Z. (2024). IHH-GLI-1-HIF-2[alpha] signalling influences hypertrophic chondrocytes to exacerbate osteoarthritis progression. J Orthop Translat.

[33] Engsig M.T., Chen Q.J., Vu T.H., Pedersen A.C., Therkidsen B., Lund L.R., Henriksen K., Lenhard T., Foged N.T., Werb Z., Delaissé J.M. (2000). Matrix metalloproteinase 9 and vascular endothelial growth factor are essential for osteoclast recruitment into developing long bones. J Cell Biol.

[34] Yang L., Tsang K.Y., Tang H.C., Chan D., Cheah K.S. (2014). Hypertrophic chondrocytes can become osteoblasts and osteocytes in endochondral bone formation. Proc Natl Acad Sci U S A.

[35] Billinghurst R.C., Dahlberg L., Ionescu M., Reiner A., Bourne R., Rorabeck C., Mitchell P., Hambor J., Diekmann O., Tschesche H., Chen J., Van Wart H., Poole A.R. (1997). Enhanced cleavage of type II collagen by collagenases in osteoarthritic articular cartilage. J Clin Invest.

[36] Gutiérrez-Martínez E., Planès R., Anselmi G., Reynolds M., Menezes S., Adiko A.C., Saveanu L., Guermonprez P. (2015). Cross-presentation of cell-associated antigens by MHC Class I in dendritic cell subsets. Front Immunol.

[37] Minond D., Lauer-Fields J.L., Cudic M., Overall C.M., Pei D., Brew K., Visse R., Nagase H., Fields G.B. (2006). The roles of substrate thermal stability and P2 and P1' subsite identity on matrix metalloproteinase triple-helical peptidase activity and collagen specificity. J Biol Chem.

[38] Tang Y., Rowe R.G., Botvinick E.L., Kurup A., Putnam A.J., Seiki M., Weaver V.M., Keller E.T., Goldstein S., Dai J., Begun D., Saunders T., Weiss S.J. (2013). MT1-MMP-dependent control of skeletal stem cell commitment via a [beta]1-integrin/YAP/TAZ signaling axis. Dev Cell.

[39] Chun T.-H., Sabeh F., Ota I., Murphy H., McDonagh K.T., Holmbeck K., Birkedal-Hansen H., Allen E.D., Weiss S.J. (2004). MT1-MMP-dependent neovessel formation within the confines of the three-dimensional extracellular matrix. J Cell Biol.

[40] Amar S., Smith L., Fields G.B. (2017). Matrix metalloproteinase collagenolysis in health and disease. Biochim Biophys Acta Mol Cell Res.

[41] Oh J., Takahashi R., Adachi E., Kondo S., Kuratomi S., Noma A., Alexander D.B., Motoda H., Okada A., Seiki M., Itoh T., Itohara S., Takahashi C., Noda M. (2004). Mutations in two matrix metalloproteinase genes, MMP-2 and MT1-MMP, are synthetic lethal in mice. Oncogene.

[42] Vu T.H., Shipley J.M., Bergers G., Berger J.E., Helms J.A., Hanahan D., Shapiro S.D., Senior R.M., Werb Z. (1998). MMP-9/gelatinase B is a key regulator of growth plate angiogenesis and apoptosis of hypertrophic chondrocytes. Cell.

[43] Lee E.R., Lamplugh L., Kluczyk B., Leblond C.P., Mort J.S. (2009). Neoepitopes reveal the features of type II collagen cleavage and the identity of a collagenase involved in the transformation of the epiphyses anlagen in development. Dev Dyn.

