# Supplementary material for: The Interstitial Collagenases MMP13 and MMP14 Are Dispensable for the Early Onset of Skin Morphogenesis but Modulate Endochondral Ossification
Source: Am J Pathol. 2026 May 15;196(8):1722–34. doi: 10.1016/j.ajpath.2026.04.018 (PMC13494082; doi:10.1016/j.ajpath.2026.04.018)
Supplement: Supplementary file 1 [file mmc1.pdf]

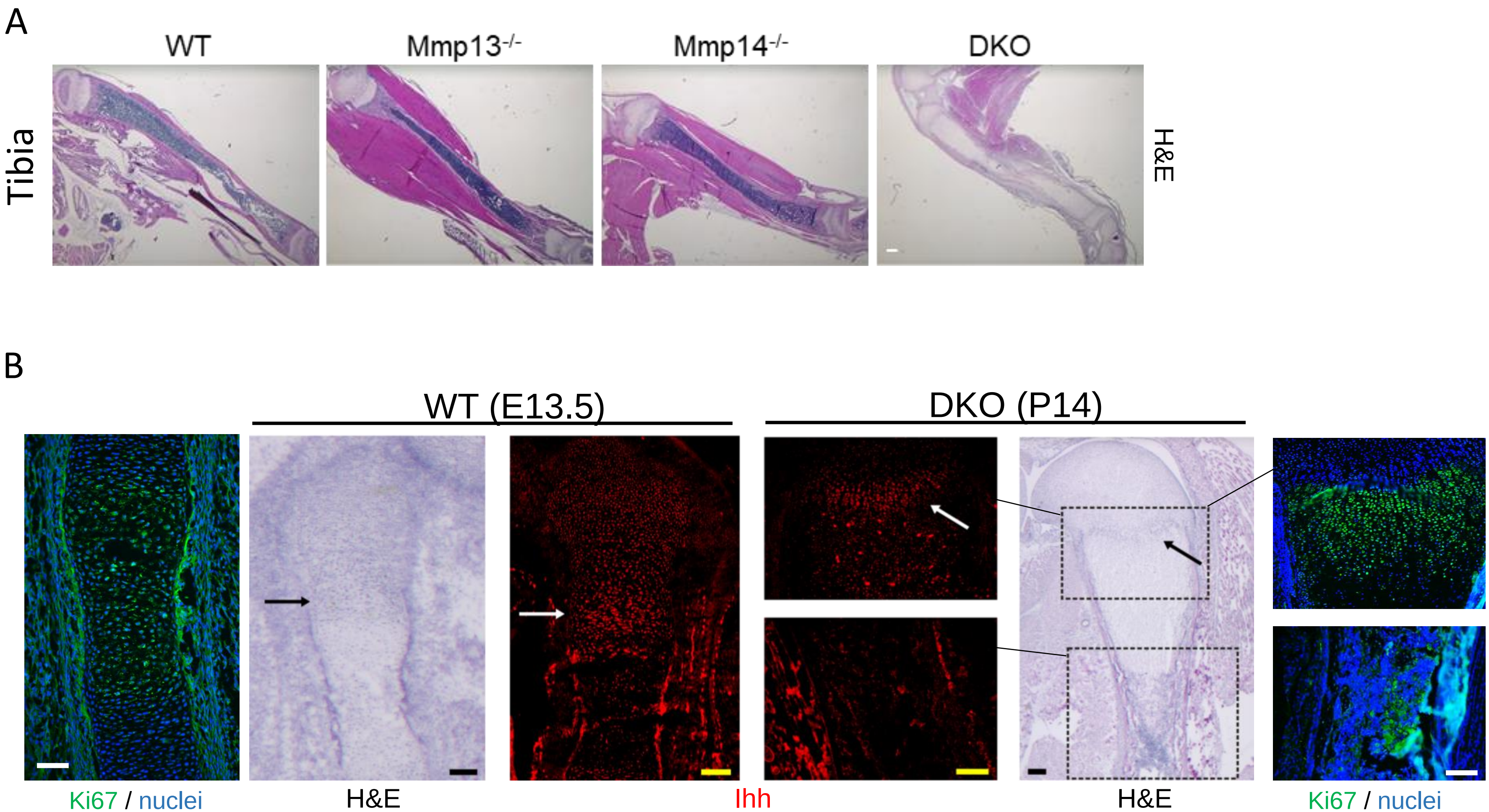
A
WT
Mmp13-/-
Mmp14-/-
DKO
Tibia
H&E
B
WT (E13.5)
DKO (P14)
Ki67 / nuclei
H&E
Ihh
H&E
Ki67 / nuclei

Figure S1
