# Supplementary material for: The Interstitial Collagenases MMP13 and MMP14 Are Dispensable for the Early Onset of Skin Morphogenesis but Modulate Endochondral Ossification
Source: Am J Pathol. 2026 May 15;196(8):1722–34. doi: 10.1016/j.ajpath.2026.04.018 (PMC13494082; doi:10.1016/j.ajpath.2026.04.018)
Supplement: Supplementary file 2 [file mmc2.pdf]

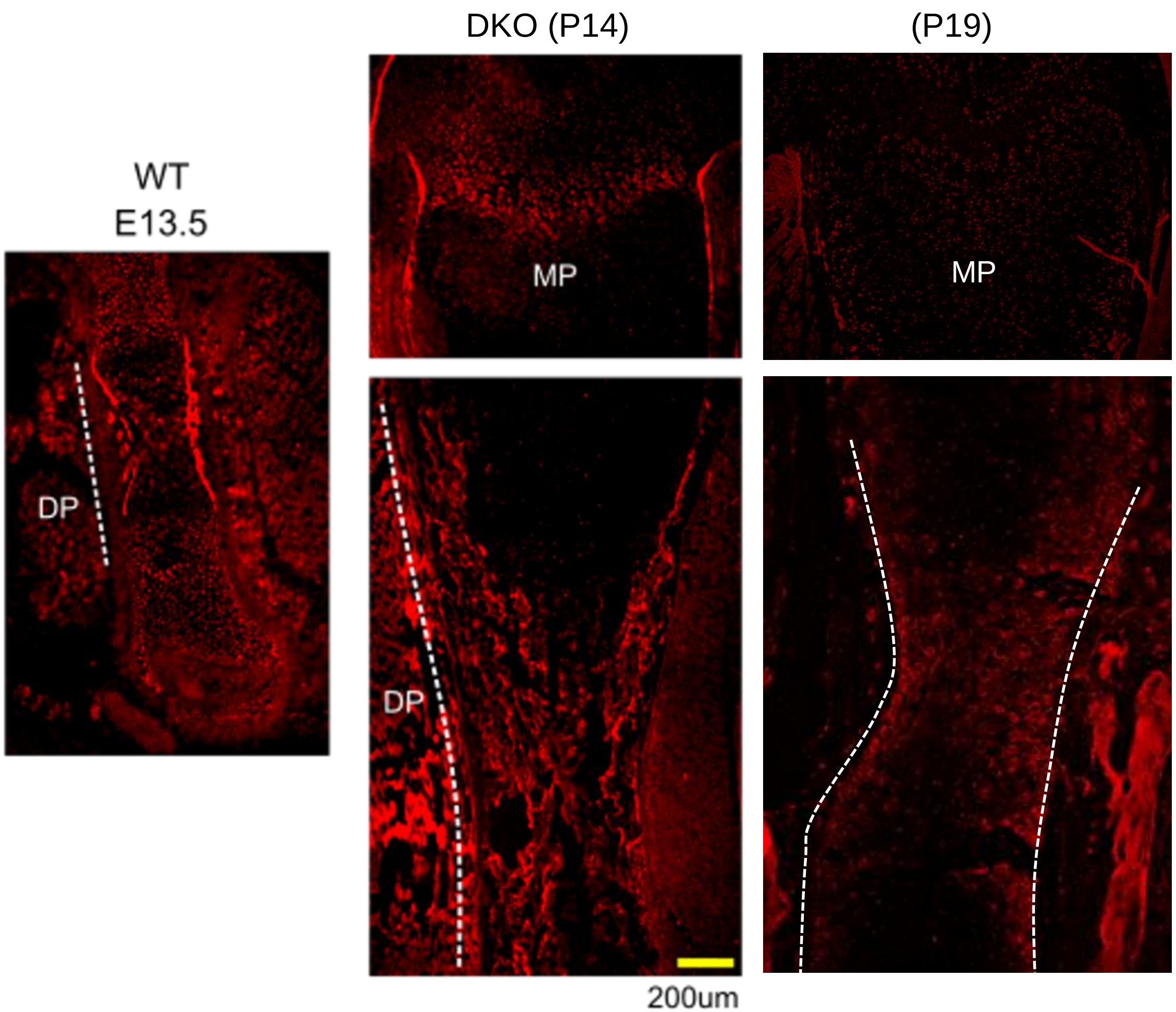

Figure S2
