# Supplementary material for: The Interstitial Collagenases MMP13 and MMP14 Are Dispensable for the Early Onset of Skin Morphogenesis but Modulate Endochondral Ossification
Source: Am J Pathol. 2026 May 15;196(8):1722–34. doi: 10.1016/j.ajpath.2026.04.018 (PMC13494082; doi:10.1016/j.ajpath.2026.04.018)
Supplement: Supplementary file 3 [file mmc3.pdf]

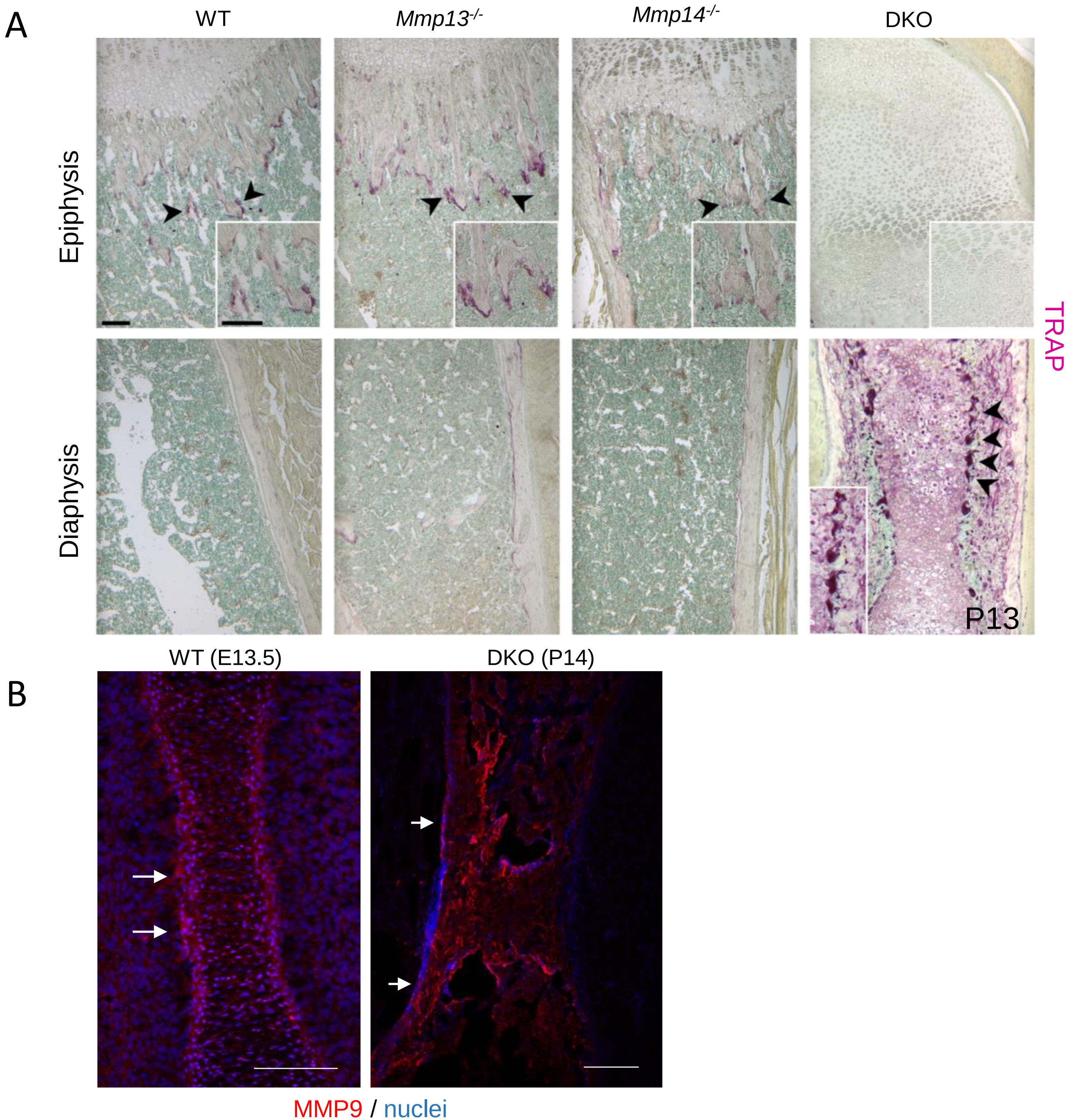
A
WT
Mmp13-/-
Mmp14-/-
DKO
Epiphysis
Diaphysis
TRAP
P13
B
WT (E13.5)
DKO (P14)
MMP9 / nuclei

Figure S3
